# Advancing Energy Storage and Catalysis with Novel Nanomaterials

**DOI:** 10.3390/ma16196425

**Published:** 2023-09-27

**Authors:** Zhenyu Yang, Jinsheng Zhao

**Affiliations:** 1 School of Chemistry and Chemical Engineering, Nanchang University, Nanchang 330031, China; 2 School of Chemistry and Chemical Engineering, Liaocheng University, Liaocheng 252059, China; j.s.zhao@163.com

In the dynamic realm of materials science, novel nanomaterials possess the transformative potential to reshape various industries, ranging from energy storage to catalysis. The objective of this Special Issue, titled “Innovative Nanomaterials for Energy Storage and Catalysis”, is to facilitate the exchange of groundbreaking research and ideas related to the synthesis, characterization, and application of innovative nanomaterials.

The articles featured in this Special Issue encompass a diverse spectrum of topics, thereby showcasing the multifaceted capabilities of nanomaterials in addressing challenges within the domains of energy storage and catalysis. Noteworthy breakthroughs include the utilization of three-dimensional flower-like MoS_2_ nanosheets and TiO_2_ nanorod-coated polyethylene separators, both of which mark significant advancements in the creation of high-performance materials designed for rapid-charging lithium-ion batteries. Furthermore, a comprehensive review delves into the realm of new materials tailored for anion-selective electrodes, offering insights into a multitude of potential applications. Our Special Issue also highlights the innovative POP-Ni catalyst for CO_2_ fixation, derived from PBTP, which offers a groundbreaking approach for the ambient fixation of CO_2_ into cyclic carbonates—a notable contribution to the ongoing endeavors related to carbon capture and utilization. Additionally, we delve into the realm of CO_2_-switchable hierarchically porous zirconium-based MOF-stabilized Pickering emulsions, elucidating the prospects of recyclable and efficient interfacial catalysis through the use of advanced materials.

These contributions highlight the diverse nature of nanomaterial research, covering various aspects such as material synthesis, hierarchical organization, device fabrication, and characterization. As readers explore this Special Issue, we encourage them to discover the incredible potential inherent in nanomaterials and their pivotal role in shaping the future of energy storage and catalysis. It is our sincere hope that the articles presented here will serve as a source of inspiration, encouraging further exploration and innovation in the field of nanomaterials. Ultimately, these efforts have the potential to bring about transformative advancements in energy storage and catalysis.

